# Ligand-Dependent and Ligand-Independent Effects of Ephrin-B2–EphB4 Signaling in Melanoma Metastatic Spine Disease

**DOI:** 10.3390/ijms22158028

**Published:** 2021-07-27

**Authors:** Andras Piffko, Thomas Broggini, Christoph Harms, Ralf Heinrich Adams, Peter Vajkoczy, Marcus Czabanka

**Affiliations:** 1 Department of Neurosurgery, University Medicine Charité, D-10117 Berlin, Germany; apiffko@uchicago.edu (A.P.); p.vajkoczy@charite.de (P.V.); marcus.czabanka@kgu.de (M.C.); 2 Department of Neurosurgery, University Medical Center Hamburg-Eppendorf, D-20251 Hamburg, Germany; 3 Department of Physics, University of California at San Diego, 9500 Gilman Drive, La Jolla, CA 92093, USA; 4 Department of Neurosurgery, University Hospital, Goethe-University, D-60528 Frankfurt am Main, Germany; 5 Center for Stroke Research Berlin, Department of Experimental Neurology, University Medicine Charité, D-10117 Berlin, Germany; christoph.harms@charite.de; 6 Department of Tissue Morphogenesis, Faculty of Medicine, Max-Planck-Institute for Molecular Biomedicine, University of Münster, D-48149 Münster, Germany; ralf.adams@mpi-muenster.mpg.de

**Keywords:** spinal bone metastasis, cancer therapy, seed and soil, Ephrin-B2–EphB4

## Abstract

Tumor–endothelial cell interactions represent an essential mechanism in spinal metastasis. Ephrin-B2–EphB4 communication induces tumor cell repulsion from the endothelium in metastatic melanoma, reducing spinal bone metastasis formation. To shed further light on the Ephrin-B2–EphB4 signaling mechanism, we researched the effects of pharmacological EphB4 receptor stimulation and inhibition in a ligand-dependent/independent context. We chose a preventative and a post-diagnostic therapeutic window. EphB4 stimulation during tumor cell seeding led to an increase in spinal metastatic loci and number of disseminated melanoma cells, as well as earlier locomotion deficits in the presence of endothelial Ephrin-B2. In the absence of endothelial Ephrin-B2, reduction of metastatic loci with a later manifestation of locomotion deficits occurred. Thus, EphB4 receptor stimulation affects metastatic dissemination depending on the presence/absence of endothelial Ephrin-B2. After the manifestation of solid metastasis, EphB4 kinase inhibition resulted in significantly earlier manifestation of locomotion deficits in the presence of the ligand. No post-diagnostic treatment effect was found in the absence of endothelial Ephrin-B2. For solid metastasis treatment, EphB4 kinase inhibition induced prometastatic effects in the presence of endothelial Ephrin-B2. In the absence of endothelial Ephrin-B2, both therapies showed no effect on the growth of solid metastasis.

## 1. Introduction

Spinal metastasis leads to severe neurological symptoms due to rapid myelopathy through metastatic epidural spinal cord compression (MESCC) and destabilization of the spinal column’s osseous structures [1]. Current treatment strategies aim at surgical decompression and stabilization of the spine followed by a combination of chemo- and radiotherapy. The decisive molecular principles of spinal bone metastasis remain inadequately understood, explaining the lack of medical strategies to prevent this pathology. The dissemination of circulating tumor cells requires tumor–endothelial cell interactions in osseous organs and represents a critical feature in the metastatic cascade.

The Ephrin-B2–EphB4 pathway belongs to the largest receptor tyrosine kinase (RTK) family and mediates cellular adhesion and repulsion in various biological scenarios [2,3,4]. The physiological interaction has been shown to induce bone homeostasis, whereas its dysfunction can induce multiple myeloma [5,6]. In metastatic disease, both proteins are associated with organ-specific metastasis development, depending on the expression of both Ephrin-B2 and EphB4 on tumor- and organ-specific endothelial cells [7]. The forward (receptor) and reverse (ligand) signaling of the Ephrin-B2–EphB4 system enables this pathway to act as a “molecular switch” mediating different, sometimes contradictory, effects [4]. Hence, EphB4 signaling exerts both protumorigenic and antitumorigenic effects that are dependent on the presence or absence of Ephrin-B2. In the context of melanoma, a protumorigenic effect has been shown [7,8,9]. In an experimental melanoma metastasis model, Ephrin-B2–EphB4 interaction prevents the metastatic dissemination of melanoma cells after retrograde carotid artery injection by inducing melanoma cell repulsion from bone endothelial cells [10]. Interference with this pathway using EphB4 kinase inhibition or genetic depletion of endothelial Ephrin-B2 resulted in increased dissemination of tumor cells, diverse metastatic loci in the spine, and an early onset of locomotion deficits [10]. However, the underlying signaling mechanism is only partly understood and the potential of this pathway for therapeutic applications remains elusive. Therefore, it was the aim to further elucidate the modulation of the metastatic capability by the Ephrin-B2–EphB4 pathway in a ligand-dependent and ligand-independent retrograde carotid artery injection melanoma metastasis model and to validate its potential for future therapeutic strategies.

## 2. Results

### 2.1. Ephrin-B2-Fc Alters Metastatic Dissemination of Melanoma Cells Depending on the Presence or Absence of Endothelial Ephrin-B2

**Figure 1:**
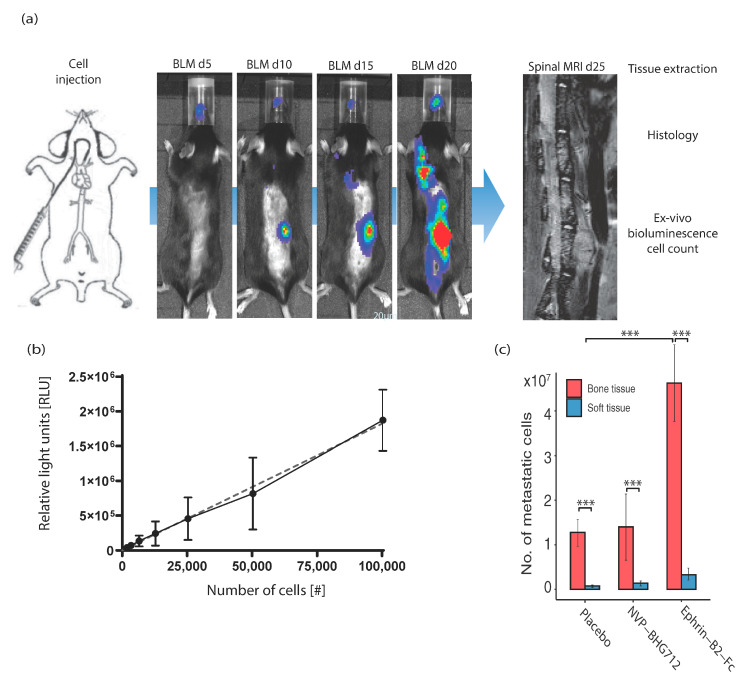
Experimental overview. (**a**) Retrograde carotid artery injection procedure followed by exemplary growth of spinal metastases in bioluminescence imaging on days 5, 15, 20, and 25 and focused spinal MRI on day 25—shown in placebo-treated *efnb2*^lox/lox^ animals. Postmortem tissue extraction was performed and the tissue was used for histological analysis and ex vivo luciferase assay for tumor cell count. (**b**) Standard curve generated after lysing 50 to 100,000 cells (*n* = 8, slope = 18.22, r^2^ = 0.8614). Standard curve used for ex vivo luciferase assay. (**c**) Significant preference of metastatic tumor cells for osseous organs independent of treatment regimen. Significant increase of metastatic cell count in osseous organs after Ephrin-B2-Fc treatment (*n* = 4, *p* = 0.001, one-way ANOVA with Dunnett’s multiple comparison test). One star (*) indicates *p* ≤ 0.05, two stars (**) indicate *p* ≤ 0.01 and three stars (***) indicate *p* ≤ 0.001.

**Figure 2:**
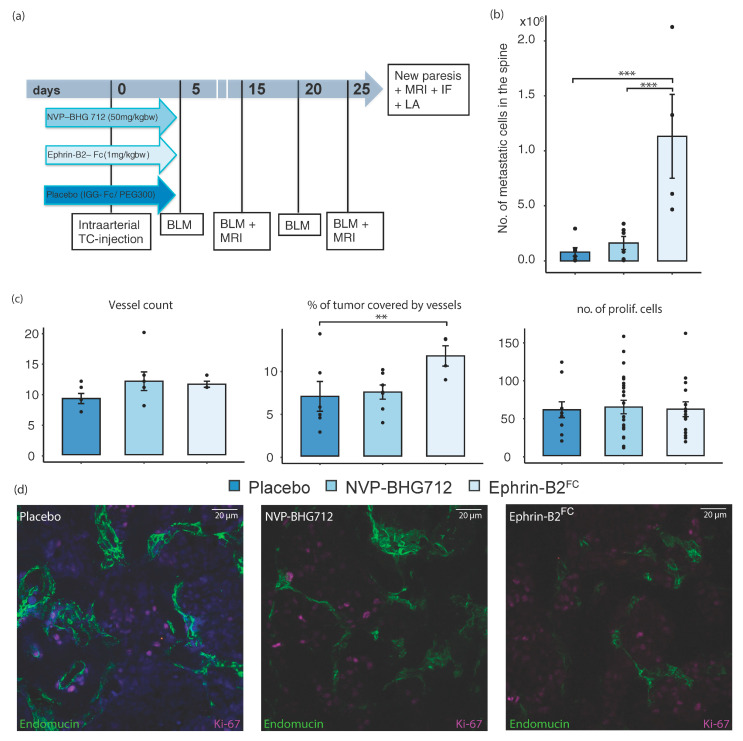
Therapy in control animals. (**a**) Timeline of experiments of NVP-BHG 712, Ephrin-B2-Fc and Placebo IGG-Fc / PEG 300 treatment in *efnb2*^lox/lox^ control mice (BLM = Bioluminescence measurements, MRI = Magnetic resonance imaging, IF = Immunofluorescence, LA = Luciferase assay) (**b**) No. of metastatic cells found in spine of Ephrin-B2-Fc treated animals significantly increased (*n* = 4, *p* = 0.002, one-way ANOVA with Dunnett’s multiple comparison test) (**c**) Number of tumor vessels per FOV was not altered significantly by therapeutic use of Ephrin-B2-Fc compared to placebo-treatment. Size of tumor vessels increased significantly upon administration of Ephrin-B2-Fc (*n* = 4, *p* = 0.0034, one-way ANOVA followed by Dunnett’s multiple comparison test). Number of proliferating cells did not show significant differences upon application of Ephrin-B2-Fc compared to placebo (*n* = 6 and 4 mice, respectively). (**d**) Representative images of confocal microscopy in spinal metastatic tumors. Staining and treatment as indicated. Mean values ± SEM for all experiments shown. One star (*) indicates *p* ≤ 0.05, two stars (**) indicate *p* ≤ 0.01 and three stars (***) indicate *p* ≤ 0.001.

The soluble EphB4-stimulating ligand Ephrin-B2-Fc or the EphB4 RTK inhibitor NVP-BHG 712 were applied during the hematogenous dissemination of metastatic melanoma cells. In this preventative therapy, the effects on tumor–endothelial cell interactions under ligand-dependent conditions, i.e., in *efnb2*^lox/lox^ control animals with physiological endothelial Ephrin-B2 expression (Figure 1a) were investigated. In this regimen, metastatic tumor cells showed a significant preference for osseous organs (forelimb, hindlimb, spine, skull) when compared to soft tissue organs (liver, kidney, lung, heart, skin), independent of therapy, with most metastatic cells found in the bones of the Ephrin-B2-Fc treatment cohort (Figure 1c). Concomitantly, the number of metastatic cells in the spine was significantly increased under Ephrin-B2-Fc treatment and remained unchanged through kinase-inhibition (Figure 2b). We identified metastatic loci with focused spinal MRI and by in vivo bioluminescence (BLM) imaging (representative images and timeline of workflow in Figure 1a). The post seeding effects such as tumor cell proliferation or tumor angiogenesis were not significantly altered. However, an increase in tumor vessels’ size was shown after ligand-treatment (Figure 2c). 

**Figure 3:**
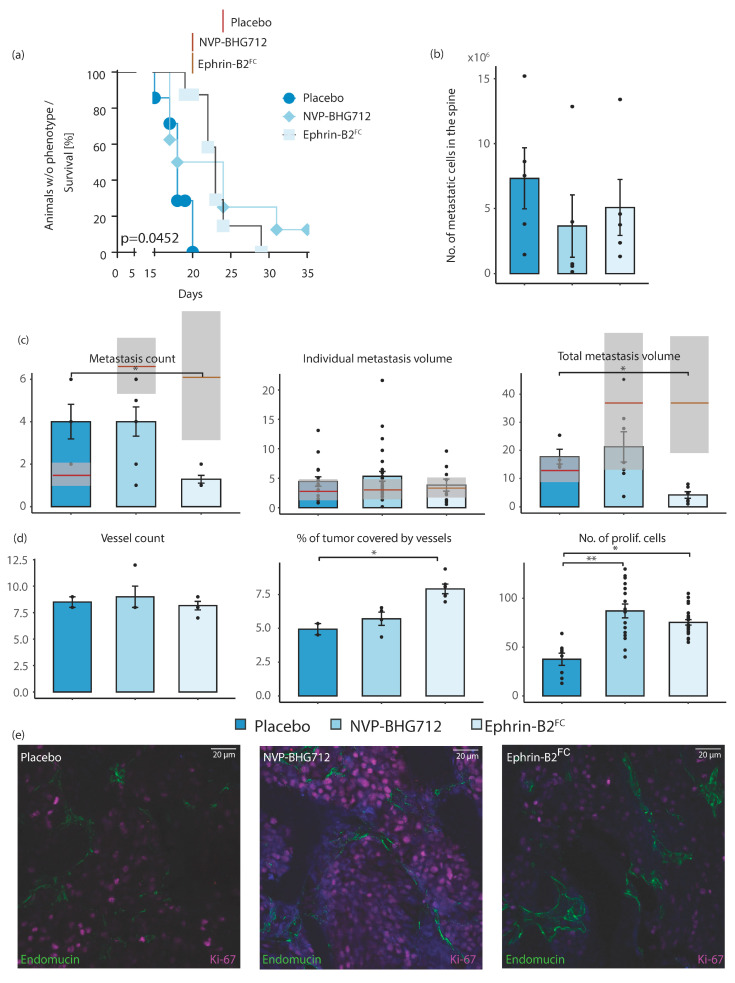
Adjuvant therapy in *efnb2*^iΔEC^ knockout animals. (**a**) Neurological deficit occurred significantly later in Ephrin-B2-Fc treated animals (median 23 days, *n* = 8 mice) compared to placebo-treated group (median placebo: 18 days, *n* = 7 mice, *p* = 0.0452, Log-rank (Mantel–Cox) test). By application of NVP-BHG 712, the time of neurological survival was not affected (*n* = 9). Significance threshold adjusted for multiple comparisons by Bonferroni method. The red bars show median survival of *efnb2*^lox/lox^ control animals receiving respective therapy published previously (placebo: 24 days, NVP-BHG 712: 20 days, Ephrin-B2-Fc: 20 days) [10]. (**b**) No significant difference in no. of spinal metastastic cells in placebo-, NVP-BHG 712- and Ephrin-B2-Fc-treated animals (*n* = 5, 4 and 6 mice, respectively). (**c**) No. of spinal metastases was significantly lower in Ephrin-B2-Fc compared to placebo-treated animals (*n* = 10 mice, *p* = 0.0371 (one-way ANOVA with Dunnett’s multiple comparison test)). Application of NVP-BHG 712 showed no significant effect on the number of spinal metastases. Individual metastasis volume was not significantly changed in both treatment groups. Total tumor volume significantly decreased after application of Ephrin-B2-Fc (*p* = 0.045). It was unaffected by the application of NVP-BHG 712 (*n* = 4 mice). Mean ± SEM for all experiments shown. The red bars show average metastasis numbers/volumes found in *efnb2*^lox/lox^ control animals receiving respective therapy published previously (shading indicates the standard deviation) [10]. (**d**) No. of tumor vessels remained unaffected. The application of Ephrin-B2-Fc significantly increased the size of tumor blood vessels (*p* = 0.0010, (one-way ANOVA with Dunnett’s multiple comparisons test)). The fraction of Ki67 positive tumor cells significantly increased after NVP-BHG 712 and Ephrin-B2-Fc treatment (*p* = 0.012). (**e**) Representative images of confocal microscopy in spinal metastatic tumors. Staining and treatment as indicated. Mean values ± SEM for all experiments shown. One star (*) indicates *p* ≤ 0.05, two stars (**) indicate *p* ≤ 0.01 and three stars (***) indicate *p* ≤ 0.001.

In endothelial cell inducible Ephrin-B2 knockout (*efnb2*^iΔEC^) mice (i.e., under ligand-independent conditions), the same preventive treatment with Ephrin-B2-Fc showed a later onset of neurological symptoms compared to placebo-treated animals (Figure 3a). The number of metastatic cells in the spine was not altered (Figure 3b), which is explained by a later time point of tumor harvest because of the longer maintenance of hindlimb locomotion. Ephrin-B2-Fc led to a significantly decreased number of metastatic loci and significantly reduced the total metastasis volume in spinal MRI 25 days post tumor cell injection (Figure 3c). The tumor cell proliferation and angiogenesis was significantly increased under Ephrin-B2-Fc treatment [Figure 3d]. EphB4 kinase inhibition did not significantly change the onset of neurological symptoms and remained without differences to placebo-treated animals in the number of metastatic cells, metastatic loci, individual and total metastasis volume (Figure 3a–d). Tumor cell proliferation was increased, whereas angiogenesis was not affected by kinase inhibition (Figure 3d).

### 2.2. EphB4 Kinase Inhibition Accelerates Metastasis Growth Depending on the Presence of Endothelial Ephrin-B2

**Figure 4:**
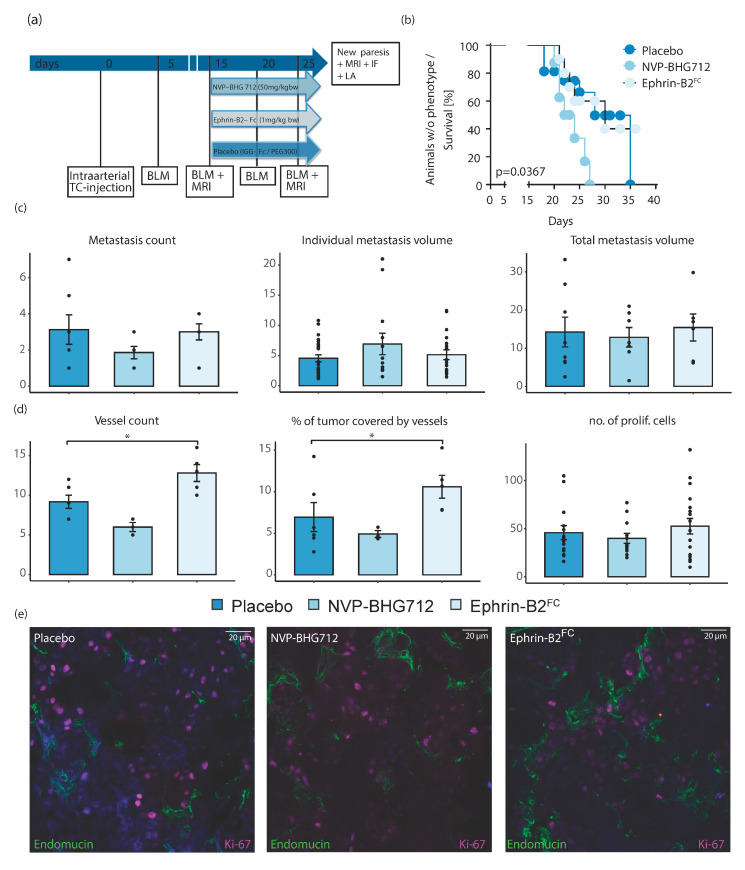
Post tumor formation therapy in control animals. (**a**) Timeline of experiments of NVP-BHG 712, Ephrin-B2-Fc and Placebo IGG-Fc / PEG 300 treatment in *efnb2*^lox/lox^ control mice (BLM = Bioluminescence measurements, MRI = Magnetic resonance imaging, IF = Immunofluorescence, LA = Luciferase assay) (**b**) Animals treated with NVP-BHG 712 showed significantly earlier signs of neurologic symptoms when compared with the placebo group (median NVP-BHG 712: 23 days, *n* = 7, *p* = 0.035, Log-rank (Mantel–Cox) test). Significance threshold adjusted for multiple comparisons by Bonferroni method. (**c**) Number of spinal metastases was unaffected by applying therapeutics. Mean individual and total tumor volume was also unaffected by therapy. Mean ± SEM for all experiments shown. (**d**) No significant proliferation differences were observed under post-tumor treatment in *efnb2*^lox/lox^ mice. Statistically significant increase in the number of tumor blood vessels of Ephrin-B2-Fc treated group (*n* = 7, 5, respectively, *p* = 0.023) and size of tumor blood vessels (*n* = 6, *p* = 0.008 (one-way ANOVA with Dunnett’s multiple comparison test)). (**e**) Representative images of confocal microscopy in spinal metastatic tumors. Staining and treatment as indicated. Mean ± SEM for all experiments shown. One star (*) indicates *p* ≤ 0.05, two stars (**) indicate *p* ≤ 0.01 and three stars (***) indicate *p* ≤ 0.001.

Ephrin-B2-Fc and NVP-BHG 712 were injected after solid metastasis development in the spinal bone under ligand-dependent conditions to mimic a therapeutic translational approach (Figure 4a). EphB4 kinase inhibition significantly shortened the time until neurological symptoms developed (Figure 4b). No alterations of metastatic loci, number of metastatic cells, individual or total metastasis volume were identified (Figure 4c,d). Ephrin-B2-Fc did not affect neurological symptoms, the number of metastatic loci or metastatic cells, individual and total metastasis volume and tumor cell proliferation (Figure 4b–d). The number and size of tumor vessels were significantly increased under the Ephrin-B2-Fc application, indicating a potential pro-angiogenic effect in the therapeutic setting (Figure 4d).

**Figure 5:**
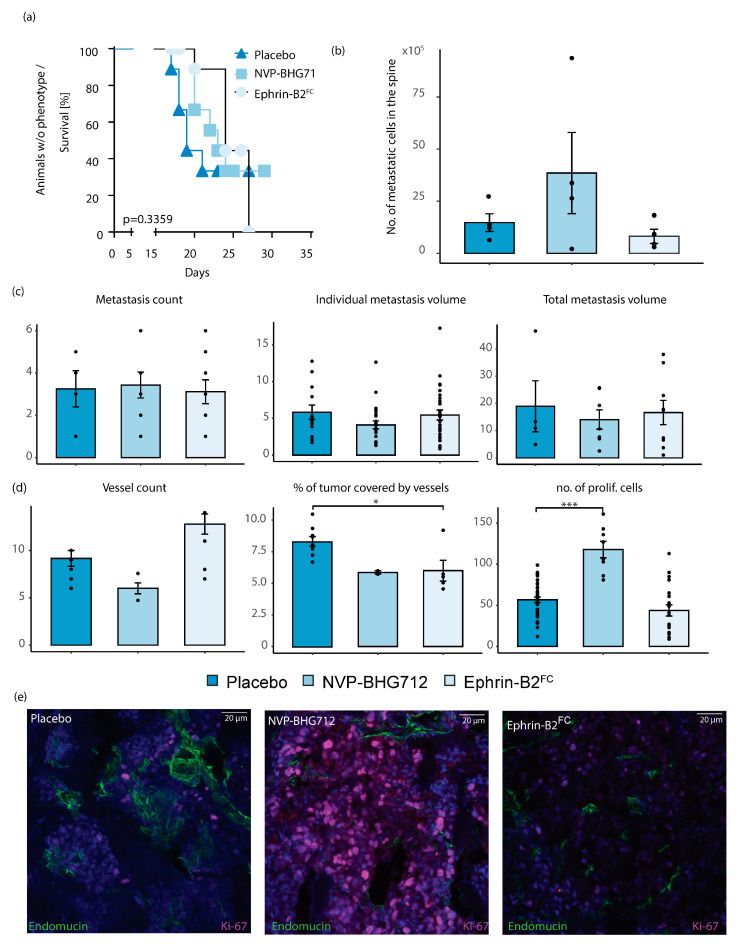
Post tumor formation therapy in *efnb2*^iΔEC^ knockout animals. (**a**) Median time until the neurological deficit was 19 days in the placebo-treated group (*n* = 9). No significant change detected after application of Ephrin-B2-Fc or NVP-BHG 712 (*n* = 11, 9, respectively, Log-rank (Mantel–Cox) test). Significance threshold adjusted for multiple comparisons by Bonferroni method. (**b**) Number of metastatic spinal cells in the placebo-treated group (*n* = 4) was not significantly affected by Ephrin-B2-Fc (*n* = 4) or NVP-BHG 712 (*n* = 4). (**c**) Mean number of spinal metastases remained unchanged (placebo *n* = 9, NVP-BHG 712 *n* = 11, EB2-Fc *n* = 9). No significant changes in mean individual and total tumor volume under therapy (One-way ANOVA with Dunnett’s multiple comparisons test, mean ± SEM for all experiments shown). (**d**) No. of tumor vessels was not significantly changed by therapy. Treatment with Ephrin-B2-Fc significantly reduced % of tumor area covered by vessels (*n* = 4, *p* = 0.0130). Tumor cell proliferation increased through NVP-BHG 712 administration (*n* = 2) and remained unaffected by Ephrin-B2-Fc administration (*n* = 5, one-way ANOVA with Dunnett’s multiple comparison test). (**e**) Representative images of confocal microscopy in spinal metastatic tumors. Staining and treatment as indicated. Mean ± SEM for all experiments are shown. One star (*) indicates *p* ≤ 0.05, two stars (**) indicate *p* ≤ 0.01 and three stars (***) indicate *p* ≤ 0.001.

Under endothelial Ephrin-B2 depletion, there were no significant differences in the neurological symptoms, metastatic loci, individual and total metastasis volume and tumor angiogenesis between either therapeutic group compared to placebo treated animals (Figure 5a–d). An increase in tumor cell proliferation was seen under EphB4 kinase inhibition (Figure 5d).

## 3. Discussion

Here, we demonstrate that the effects of the Ephrin-B2–EphB4 interaction on the metastatic dissemination of circulating melanoma cells depend on intact forward and reverse signaling. Interference with reverse signaling using soluble Ephrin-B2-Fc or the inhibition of EphB4-mediated forward signaling using kinase inhibition led to an earlier manifestation of neurological symptoms and increased metastatic loci, indicating a prometastatic effect. In the absence of endothelial Ephrin-B2, Ephrin-B2-Fc prolongs the time window until neurological symptoms and reduces the size and number of spinal metastatic loci. In the treatment of solid spinal metastasis, EphB4 kinase inhibition shortened the time until neurological deficits, highlighting a protumorigenic effect in the presence of endothelial Ephrin-B2. Under endothelial Ephrin-B2 depletion, neither Ephrin-B2-Fc nor the kinase inhibition influenced the growth of spinal metastases. 

Recent data suggests that the effect of EphB4 on the metastatic process in malignant melanoma is highly dependent on the absence or presence of its preferred ligand Ephrin-B2, and its effects are primarily related to tumor cell dissemination and not post-seeding processes like the growth of solid metastases [3,7]. NVP-BHG 712 has been evaluated in vitro and in vivo to inhibit intracellular EphB4 kinase activity efficiently [10]. Consequently, applying NVP-BHG 712 during the metastatic dissemination of melanoma cells inhibits EphB4-mediated forward signaling in tumor cells. In contrast, Ephrin-B2-Fc, a dimeric extracellular domain (ECD) of Ephrin-B2, has been associated with opposing effects on EphB4, leading to either the activation or downregulation of EphB4 [11]. Nevertheless, the application of the soluble antibody fragment Ephrin-B2-Fc leads to the interruption of Ephrin-B2-mediated reverse signaling because it is not cell-bound, representing a tool to prevent reverse signaling to, e.g., endothelial cells [12]. In the setting of *efnb2*^lox/lox^ control mice, endothelial expression of Ephrin-B2 is present. In this context, NVP-BHG 712 has been shown to increase spinal metastasis and metastasis-induced neurological deficits when applied during the hematogenous metastatic dissemination of melanoma cells [10]. Ephrin-B2-Fc induces a comparable effect by increasing spinal metastasis when applied during the dissemination of circulating tumor cells. In vitro experiments demonstrate that Ephrin-B2-Fc leads primarily to activation of cell-bound EphB4 [13]. However, the promigratory effects of EphB4 in A375 melanoma cells are independent of forward signaling, underlining the importance of reverse signaling for migratory processes [3,7], which are decisive during metastatic dissemination [12]. 

Interrupted reverse signaling to endothelial cells may translate into increased adhesion of melanoma cells to bone endothelium comparable to observations under endothelial Ephrin-B2 depletion [10]. The significant increase in the number of spinal metastases, while individual metastasis volume remained unaffected, implies that seeding mechanisms have actively been affected and that the increased extravasation of circulating tumor cells has taken place [10]. In contrast, *efnb2*^iΔEC^ mice are characterized by endothelial-specific depletion of Ephrin-B2 [14]. Under these conditions, Ephrin-B2-Fc leads to a reduction of metastatic loci and a prolonged time window until neurological deficits occur if applied during the dissemination of tumor cells. Consequently, antimetastatic effects are observed, contradicting the prometastatic results in *efnb2*^lox/lox^ control animals. Under endothelial Ephrin-B2 depletion, the barrier function of Ephrin-B2–EphB4 is severely impeded through the lack of endothelial reverse signaling [14]. Application of soluble Ephrin-B2-Fc in this setting seems to reestablish this repulsive pathway leading to a reduction of osseous spinal metastatic loci. The activation of cell-bound EphB4 resulting in increased cellular repulsion from bone endothelial cells might be responsible for this observation. The overexpression of EphB4 in the presence of endothelial Ephrin-B2 has been shown to induce similar effects by leading to an increase in melanoma cell repulsion from the endothelial cells, translating into a reduced number of spinal metastatic loci and a prolongation of the time window until neurological deficits occur [10]. Interestingly, the application of NVP-BHG 712 under endothelial Ephrin-B2 depletion did not result in antimetastatic effects, which might be explained by the missing activation of tumor cell-bound EphB4 in the absence of endothelial Ephrin-B2.

After spinal metastases are established, only NVP-BHG 712 alters metastasis growth by shortening the time window until the neurological deficits occur. The increase of the metastasis growth rate is demonstrated by a comparable size of total and individual metastasis volume between groups. Harvesting of the spinal metastasis was performed after neurological deficits occurred. Therefore, the tumor cells had to grow faster under NVP-BHG 712 treatment to reach a comparable volume as they were harvested at a significantly earlier time point. However, the increased rate of tumor growth was not paralleled by differences in tumor angiogenesis. 

Ephrin-B2–EphB4 interactions have also been implicated in the interaction between tumor cells and osteoblasts in bone metastasis development [5]. Potentially, interference with this interaction might play a role in the observed prometastatic effects under NVP-BHG 712 treatment. Interestingly, under endothelial Ephrin-B2 depletion, the protumorigenic effects of NVP-BHG 712 did not progress, even though we could demonstrate an increased number of proliferating tumor cells. This indicates that the endothelial Ephrin-B2–EphB4 interaction contributes to metastasis growth during the development of solid spinal metastasis. The underlying mechanisms remain elusive and require further analysis to elucidate the cellular consequences of EphB4 inhibition in spinal melanoma metastatic disease. In this regard, Ephrin-B2-Fc neither affected the neurological symptoms nor the individual or total metastasis volume and therefore remained ineffective independent from endothelial Ephrin-B2 expression. It seems that Ephrin-B2-Fc exerts only minor effects when melanoma cells have reached the metastatic niche and are developing into a solid lesion. Different interactions between varieties of cells that express Ephrin-B2 on their surface (e.g., osteoblasts, endothelial cells) might be responsible for this phenomenon.

In conclusion, the Ephrin-B2–EphB4 pathway is primarily involved in mediating tumor-endothelial cell interactions during metastatic dissemination of circulating melanoma cells. Both forward and reverse signaling and ligand-dependent and -independent mechanisms have to be considered when exploiting this pathway for preventing tumor cell extravasation to the bone. The stimulation of intact Ephrin-B2–EphB4 communication might represent a more promising approach for this purpose compared to interruptive strategies using Ephrin-B2-FC or NVP-BHG 712

## 4. Materials and Methods

### 4.1. Cell Line and Cell Culture

Luciferase-expressing B16-luc mouse melanoma cells (ATCC^®^ CRL-6323™) cells were infected with a FFLUC-eGFP-Puro vector as described before [9,15]. Cells were routinely maintained in DMEM-high glucose medium supplemented with 10% FBS, 50 units/mL penicillin, 50 μg/mL streptomycin and 5 μg/mL puromycin in a humidified incubator at 37 °C and 5% CO_2_. 

### 4.2. Animal Preparation

Adult tamoxifen-inducible, EC-specific Ephrin-B2-knockout (*efnb2*^iΔEC^) mice and *efnb2*^lox/lox^ littermates were used for the study (C57/Bl6J.Alb.EB2.CDH5creERT2 strain) [16]. Both groups were injected i.p. with tamoxifen (75 mg/kg bw) to activate Cre-induced endothelial Ephrin-B2-knockout in *efnb2*^iΔEC^ mice (according to Jackson Laboratory protocol, Heffner, 2011). All mice were submitted to a 7-day resting period before inclusion in the experiment. After tumor cell injection mice were checked daily to analyze behavior and symmetrical hind limb movement. 

### 4.3. Retrograde Carotid Artery Injection

A retrograde intraarterial injection method was performed as described previously [15]. Briefly, adult mice (*efnb2*^iΔEC^ and *efnb2*^lox/lox^ littermates) were anesthetized, and a longitudinal skin incision was performed on the left region of the neck to expose the common carotid artery. The artery was temporarily ligated and a catheter (0.8 mm Ø) was retrogradely inserted and fixed. B16-luc mouse melanoma cells (1 × 10^5^ cells suspended in 100 μL DMEM) were slowly injected retrogradely into the aortic arch, followed by 100 µL 0.9% NaCl. The artery was permanently ligated, the catheter was removed and the skin was sutured. 

### 4.4. Treatment Regimens

#### 4.4.1. Dissemination (Pre-Tumor) Treatment

Ephrin-B2-Fc treatment cohort (*efnb2*^iΔEC^ and *efnb2*^lox/lox^) received 4 i.v. injections of 100 μL Ephrin-B2-Fc (R&D Systems, Minneapolis, MI, USA, 1 mg/kg bw) on days −5, −2, 1 and 4. NVP-BHG 712 treatment cohort (*efnb2*^iΔEC^ and *efnb2*^lox/lox^) received 100 μL of NVP-BHG 712 (Sigma Aldrich, St. Louis, MO, USA, 50 mg/kg bw) i.p. for 8 consecutive days, starting on day 5. 

#### 4.4.2. Metastasis (Post-Tumor) Treatment

Ephrin-B2-Fc treatment cohort (*efnb2*^iΔEC^ and *efnb2*^lox/lox^) received 4 i.v. injections of 100 μL Ephrin-B2-Fc (R&D Systems, Minneapolis, MI, USA, 1 mg/kg bw) on days 12, 15, 18 and 21. NVP-BHG 712 treatment group (*efnb2*^iΔEC^ and *efnb2*^lox/lox^) received 100 μL of NVP-BHG 712 (Sigma Aldrich, St. Louis, MO, USA, 50 mg/kg bw) i.p. for 8 consecutive days, starting on day 13. Placebo group received either one injection containing 4.35 μL of IgG-Fc (i.v.) every three days, or 100 μL PEG 300 (i.p.) for 8 consecutive days.

### 4.5. In Vivo Bioluminescence Imaging 

Bioluminescence imaging was performed on postoperative days 5, 15, 20 and 25 using the IVIS Lumina II (Caliper LS, Hopkinton, MA, USA). Mice were anesthetized using 2% isoflurane. D-luciferin (Caliper LS, Hopkinton, MA, USA) solution (30 mg/mL) was injected i.p. as described in the manufacturers protocol (10 μL/g bw). A dorsal and a ventral image were obtained, each with an exposure time of 5 min. 

### 4.6. In Vivo Magnetic Resonance Imaging

MRI scans were performed regularly on day 15 and 25 and whenever any gait or movement abnormalities of the hind limbs occurred. A 7-Tesla rodent MRI scanner (BioSpec 70/20 USR, Brucker, Billerica, MA, USA) with a 16cm horizontal bore magnet and a 1H-RF-Volumeresonater (72 mm) was used. The H-resonance frequency was 300 MHz, the maximum gradient strength was 300 mT/m. Mice were placed on a heating mat and continuously anesthetized using 1.5–2% isoflurane. Paravision 6 (Brucker, Billerica, MA, USA) was used to generate sagittal T2-weighted images-10 sagittal slices of 2 mm (0.5 × 30 × 30 mm). Images were assessed using Analyze 11.0 software (Mayo Clinic, Rochester, MI, USA). Tumor area was marked manually in all slices and number of tumors, total and mean metastatic volumes were calculated.

### 4.7. Behavior Analysis

Mice were screened daily for any gait or movement abnormalities of the hind limbs. As described previously hind limb paresis occurs abruptly in this experimental mouse model [10].

### 4.8. Tissue Homogenization 

Animals were sacrificed at the given time points. All organs were immediately frozen in −50 °C isopentane and transferred to −80 °C for long-term storage. Osseous organs were pulverized using pestle and mortar under continuous cooling with liquid nitrogen (particle size 1–2 mm). Powder and soft tissue organs were transferred to individual gentle macs tubes (Miltenyi Biotec, Bergisch Gladbach, Germany) and homogenized using Xiril Dispomix tissue tearer (Miltenyi Biotec, Bergisch Gladbach, Germany) at 400 rpms for 15 s twice. Lysates were constantly kept on ice and consequently centrifuged at 1300× *g* for 5 min at 4 °C (Heraeus Multifuge, Thermo Fisher Scientific, Waltham, MA, USA). Supernatant was transferred and utilized for in vitro luminometry. 

### 4.9. In Vitro Luminometry for Cell Count

A standard calibration curve was generated using a Tecan 200M spectrometer (Tecan, Männedorf, Switzerland) after addition of 60 µL Bright-Glo luciferase substrate to 30 microliters of organ lysate supernatant in a black flat bottom 96 well plate. The measurement and quantification of the relative number of photons (Relative Light Units = RLU) was measured for three seconds as described in the manufacturers kit (Promega, Fitchburg, WI, USA). Specific organ metastasis to the spine was analyzed by calculating the approximate number of bioluminescent metastatic tumor cells after termination of the study. For this, a standard calibration curve was created by using know numbers of light-emitting B16-luc tumor cells and extrapolating the number of relative light (RLUs) generated by tumor cell lysates (Resulting curve slope 18.22 ± 0.7540 (95% confidence interval). The number of tumor cells was calculated for every individual organ including the spinal cord.

### 4.10. Immunohistochemical Staining and Processing of Spines

Murine spines were immediately fixed in 4% (wt/vol) PFA for 4 h at 4 °C. Decalcification was carried out using 0.5M EDTA at 4 °C under constant agitation for 96 h. For cryoprotection, decalcified spines were immersed in 20% (wt/vol) succrose and 2% (wt/vol) polyvinylpyrrolidone (PVP, Sigma Aldrich, St. Louis, MO, USA)) for 24 h. The freezing was carried out in a gelatin-based freezing solution containing 8% (wt/vol) gelatin, 20% (wt/vol) sucrose and 2% (wt/vol) PVP. Sagittal sections of 200 μm were prepared by using a Cryotome (Microm HM 560—Thermo Fisher Scientific, Waltham, MA, USA). Sections were permeabilized using 0.3% Triton X-100 for 10 min at RT, unspecific binding was precluded by blocking in 1% (wt/vol) casein/PBS for 30 min at RT. Incubation with primary antibodies was carried out in 1% (wt/vol) casein/PBS for 2 h at RT, samples were then washed three times with PBS and incubated with appropriate secondary antibodies for 1 h at RT (1:400). Nuclei were counterstained with DAPI for 10 min. Slides were embedded after thorough washing in Immu-Mount (Thermo Fisher, Waltham, MA, USA), mounted with glass cover slips and kept in a dark place at 4 °C. Confocal microscopy was used with tile Z-stacks of 60–70 micrometers (Z-step size 4 micrometers), generated using an oil-immersed 63× magnification in a Leica DM 2500 microscope (Leica, Wetzlar, Germany). 

### 4.11. Immunohistological Analysis

Four field of views (FOVs) were analyzed in *n* = 5 slices per tumor. The number of tumor blood vessels and their area coverage was analyzed by generating stacked images of scan z-stacks (15–16 images per stack) and manually defining regions of interests (ROIs) using freely available ImageJ software (NIH, Bethesda, MD, USA, http://imagej.nih.gov/ij/, date accessed on 6 January 2015). The number of Ki67+ proliferating cells was determined using the Cell Counter application of ImageJ in the z-stacks. Analysis of acquired data was performed as depicted in the Statistical Analysis Section.

### 4.12. Statistical Analysis

Quantitative data are given as mean ± SEM. Number of animals used is indicated in the respective figure legend. Log-rank (Mantel–Cox) test was used for the comparison of survival data; significance level was adjusted for multiple comparisons utilizing the Bonferroni method. A one-way ANOVA followed by Dunnett’s multiple comparison correction was applied for differences in multiple group comparisons. An unpaired *t*-test was applied to compare two groups. Results with *p* < 0.05 were considered significant. One star (*) indicates *p* ≤ 0.05, two stars (**) indicate *p* ≤ 0.01 and three stars (***) indicate *p* ≤ 0.001. GraphPad Prism 6 (Graphpad, La Jolla, CA, USA), RStudio 1.3.1093 (Boston, MA, USA), Adobe Illustrator (Adobe, San José, CA, USA) and Microsoft Excel (Microsoft, Seattle, WA, USA) software were used. Graphs were generated using open source R packages (ggplot2, RColorBrewer).
